# Color-Induced Aroma Illusion: Color Cues Can Modulate Consumer Perception, Acceptance, and Emotional Responses toward Cooked Rice

**DOI:** 10.3390/foods9121845

**Published:** 2020-12-11

**Authors:** Shady Afrin Jeesan, Han-Seok Seo

**Affiliations:** Department of Food Science, University of Arkansas, Fayetteville, AR 72704, USA; sjeesan@uark.edu

**Keywords:** rice, color, odor, check-all-that-apply, emotion, acceptance, willingness to eat, culture

## Abstract

Since rice is often cooked in many countries with different types of ingredients or seasonings, the surface colors of traditional rice meal items vary across cultural backgrounds. This study aimed to determine whether consumer perception, acceptance, willingness to eat, and emotional responses toward cooked rice samples could differ with their surface color cues. Milled rice was cooked with one of three food colorants: yellow, orange, and green, with milled (white) and un-milled (brown) rice cooked without colorants used as respective test and filler samples. Using a check-all-that-apply method, 98 rice consumers checked all aroma attributes they perceived by sniffing each of the four cooked-rice samples (white, yellow, orange, and green). They also rated the four samples with respect to attribute intensity, liking, emotional responses, and willingness to eat. The results showed that participants associated colored rice with specific ingredient-related aroma attributes (e.g., green color elicited sweet peas or spinach aromas). Color cues also affected ratings of attribute intensity, liking, willingness to eat, and emotional responses to cooked rice samples. In conclusion, this study provides empirical evidence that in the context of cooked rice consumption, color cues can elicit associated aromas and modulate consumer perception, acceptance, and evoked emotions to cooked rice.

## 1. Introduction

Visual cues of food or beverage items often overwhelm consumer perceptions induced by other modalities [1,2,3,4] (see also [5,6]). Color cues of either food/beverage items [1,7,8,9,10,11] or surrounding contexts of food/beverage items [12,13,14,15] have been found to play particularly important roles in consumer acceptance and eating (or purchase)-related behaviors toward the food/beverage items. For example, hedonic ratings for orange juice samples were found to differ with their color cues [7,10]. In addition, sliced apples or bell peppers were found to be more appealing under yellow lighting than under blue, green, or red lighting [14].

Earlier studies have also shown that color cues of food or beverage items alter not only “expected”, but also “perceived” attributes of such items [8,16,17,18]. One well-known example related to color-induced odor perception is Morrot et al.’s study in which participants, in describing a white wine sample using white wine-related odor descriptors, were found to use red wine-related odor descriptors when a white wine sample was artificially colored red [16]. In a more recent study by Wang and Spence [18], participants tasted three wine samples: a white wine, a rosé wine, and the white wine dyed to match the rosé wine. Even though participants found that the dyed rosé wine was different from the undyed rosé wine, they used red fruit-related descriptors for characterizing both rosé wine samples. Color cues have also been found to modulate perceived intensities of food/beverage items [19,20,21,22,23,24]. In a recent study using photographic images of carrot samples that varied in hue, saturation, and value, consumer participants responded differently with respect to the expected taste and spiciness of carrot image samples [19]. For example, red carrots when compared to the orange carrots (most familiar type) were expected to taste sourer and spicier, and purple carrots were expected to taste more bitter. Using both colorless and colored tasting solutions, Maga [20] determined the effects of color cues on perceived sensitivity to taste quality, with yellow color decreasing sensitivity to sweetness and green color increasing sensitivity to a sucrose solution. For bitter-tasting solutions (caffeine), a red color decreased bitter-taste sensitivity. The effect of color cues on perceived intensity has also been observed in odorous stimuli such as flavor solutions [21,22,23] and odorants [24] (for a review, see [25]).

Color cues are also strongly associated with emotions [26,27,28]. For example, while bright colors were found to elicit positive emotions, dark colors were found to evoke negative emotions [26]. Color–emotion associations have also been found to vary with age [29], gender [30], and cultural background [31]. For example, while yellow in the U.S. is usually associated with warmth, in France, it is associated with infidelity [31]. Recent studies have highlighted that color cues, whether intrinsic (e.g., surface colors of food or beverage samples) or extrinsic (e.g., colors of packaging) factors, can affect emotional responses to target samples [32,33,34]. For example, Chonpracha et al. [34] showed that darker green salads elicited higher intensities of positive emotions than paler green salads, with variations in eight emotional responses such as “active”, “bored”, “energetic”, “feel wellness”, “good”, “healthy”, “interested”, and “satisfied” [34]. Food/beverage-evoked emotions have been found to modulate consumer acceptability and purchase-related behavior toward food/beverage products [33,34,35,36].

Although more than 3.5 billion people consume rice as their staple food [37], there are regional differences in rice varieties cultivated and consumed all over the world. Rice cooking processes, eating patterns, dietary habits, and even eating methods differ with cultural background (for a review, see [38]). As a result of cultural differences in eating patterns and added ingredients, the surface color of cooked rice varies with cultural backgrounds. Since Korean and Japanese people often prepare cooked rice without specific ingredients or seasonings, white is the most common color of cooked rice consumed in Korea and Japan. Yellow-colored cooked rice is traditionally consumed in Bangladesh, India, Nepal, Sri Lanka, Indonesia, Iran, Afghanistan, Cuba, Peru, South Africa, and Caribbean countries. People in those countries generally prepare cooked rice with the addition of turmeric, saffron, or annatto, bringing a yellow color to their cooked rice meals. Orange-colored cooked rice meals are also observed in Bangladesh (e.g., Jorda), Nigeria (e.g., Jallof rice), and Mexico (e.g., Spanish rice). Finally, green-colored cooked rice meals (e.g., spinach rice) are often consumed in some other countries such as Spain and India.

As shown by the above examples, surface colors of typically consumed cooked-rice meals differ with cultural backgrounds. By building on previous findings related to the effects of color cues on consumer perception, acceptance, and emotional responses toward odors, tasting substances, and food/beverage items, this study aimed at determining whether such effects of color cues can exhibit in cooked rice samples with four specific aims. First, this study aimed at determining whether participants perceive aroma attributes that differ with surface colors of cooked rice samples (e.g., tomato aromas in response to red-colored cooked rice). Second, this study tested whether surface colors affect attribute intensities and hedonic impressions of cooked rice samples. Third, this study also tested whether emotional responses to cooked rice samples vary with respect to their surface colors. Finally, this study aimed to determine whether participants’ willingness to consume cooked rice samples differs with respect to their surface colors. Most studies related to the effect of color cues in sensory studies have been conducted using liquid-based matrices such as taste solutions, beverages, or drinks [7,9,10,13,16,17,18,20,21,22,23], which is probably because it would be easier for researchers to manipulate color cues in such matrices than in semi-solid or solid-based matrices. Therefore, this study using a solid-based matrix would bring additional merit testing whether the effect of color cues shown in liquid-based matrices are also exhibited in other types of matrixes.

## 2. Materials and Methods

### 2.1. Participants

Ninety-eight participants (30 males and 68 females) aged from 19 to 78 years (mean age ± standard deviation (SD) = 37.6 ± 13.9 years) were recruited through a consumer profile database provided by the University of Arkansas Sensory Science Center (Fayetteville, AR, USA). This sample size was within the range of 40–100 consumers as recommended by Gauchla and Rutenbeck [39] for consumer testing. All participants (51 Caucasians, 27 Asians, 16 Hispanics, 2 African-Americans, 1 Native American, and 1 other) self-reported no clinical history of major disease, eating disorder, or food allergies that might influence their sensory perception or emotional state. In addition, all self-reported having eaten cooked rice meals at least twice a week. Participants self-rated themselves as to whether they were neither full nor hungry (mean ± SD = 2.47 ± 0.72) on a 5-point category scale ranging from 1 (very hungry) to 5 (very full) and rated whether they were in a good mood (mean ± SD = 4.85 ± 0.58) on a 5-point category scale ranging from 1 (very bad) to 5 (very good).

This study was conducted per the Declaration of Helsinki for studies on human participants, and its protocol was approved by the Institutional Review Board of the University of Arkansas (Fayetteville, AR, USA). Prior to participation, a written informed consent was obtained from each participant.

### 2.2. Rice Samples and Preparation

A total of five cooked-rice samples, four test samples and one filler, were served to each participant. For the four test samples, milled long-grain white rice (unknown cultivar; Mahatma, Riviana Foods Inc., Houston, TX, USA) was purchased from a local grocery store (Fayetteville, AR, USA) and un-milled long-grain rice, i.e., brown rice, (unknown cultivar; Great Value, Bentonville, AR, USA) was purchased for the filler sample.

**Figure 1:**
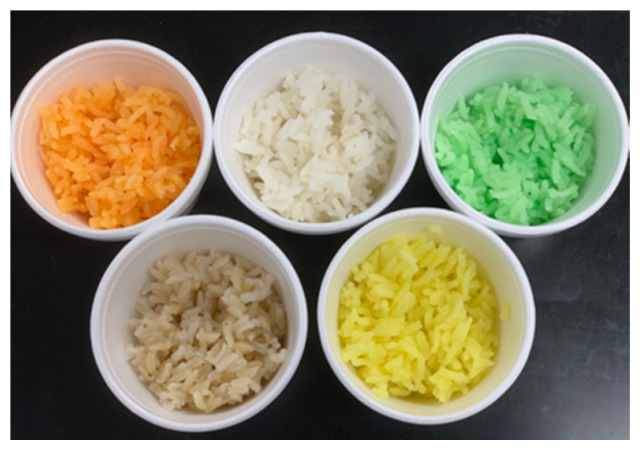
Cooked rice samples used in this study. Four test samples (in a clockwise direction from the top left corner: orange, white, green, and yellow) and one filler (in the bottom left corner: brown) were served.

The surface colors of the four test samples of cooked rice varied: white, yellow, orange, and green (Figure 1). More specifically, for the white color sample, 350 g of milled-rice grain product were rinsed and cooked with 700 mL of water (Clear Mountain Spring Water, Taylor Distribution, Heber Springs, AR, USA) in an electronic rice cooker (JNP-1500-FL, Tiger Corporation U.S.A., Torrance, CA, USA). For the yellow, orange, or green color sample, 350 g of milled-rice grains were rinsed and cooked with water (700 mL) containing yellow (200 mg), orange (200 mg), or green (100 mg) food-colorant powder (CK products, Elkhart, IA, USA).

For the filler sample, 350 g of un-milled rice grain product were rinsed and cooked with water in an electronic rice cooker, using a 1:2.5 (*w*/*w*) rice-to-water ratio. Optimum rice-to-water ratios used in this study were determined based on preliminary testing of each rice product.

### 2.3. Check-All-That-Apply (CATA) Question Ballots

The check-all-that-apply (CATA) method [40] was used to characterize variation in sensory attributes of and emotional responses to cooked rice samples as a function of surface color. When using CATA question ballots, participants are asked to select all terms that they consider appropriate to characterize the sensory or emotional attributes of each test sample. Previous studies had proven that this method was effective in characterizing sample differences with respect to sensory attribute or emotional response [41,42,43,44,45,46,47,48]. Most consumer participants were found to accurately use the CATA terms for describing sensory attributes that they perceived in test samples [45], and their results were also found to be similar to those obtained from descriptive sensory analysis using trained panel [46,47,48]. Jaeger et al. [45] also found that most participants did not select specific terms when they did not perceive the corresponding sensory attributes in the test samples. These findings suggest that the CATA method can be useful for characterizing variations in sensory or emotional attributes with surface color cues of cooked rice samples.

Based both on previous sensory studies related to cooked rice [42,49,50] and our preliminary study using colored rice samples, the sensory attribute-related CATA question (hereafter abbreviated “sensory CATA”) included 15 sensory attributes (especially aroma-related descriptors): “cilantro”, “cloves”, cooked rice”, “curry”, “floral”, “ginger”, “oil”, “onion”, “popcorn”, “red peppers”, “saffron”, “spinach”, “sweet peas”, “tomato”, and “turmeric” (see Table 1). The emotion-attribute-related CATA question (hereafter abbreviated “emotion CATA”) included 39 emotion-related terms of the EsSense Profile^®^ [51]: “active”, “adventurous”, “affectionate”, “aggressive”, “bored”, “calm”, “daring”, “disgusted”, “eager”, “energetic”, “enthusiastic”, “free”, “friendly”, “glad”, “good”, “good-natured”, “guilty”, “happy”, “interested”, “joyful”, “loving”, “merry”, “mild”, “nostalgic”, “peaceful”, “pleasant”, “pleased”, “polite”, “quiet”, “satisfied”, “secure”, “steady”, “tame”, “tender”, “understanding”, “warm”, “whole”, “wild”, and “worried” (see Table 2). In sensory or emotion-related CATA questions, these terms were listed in alphabetical order to assist participants in finding all attributes that they wanted to check. Lee et al. [52] showed that the effect of CATA term order on consumer responses to test samples was minimal.

### 2.4. Procedure

Prior to the main test, all participants were asked to evaluate a warm-up sample to ensure they could understand all sensory terms listed on the check-all-that-apply (CATA) question ballots and become familiar with the test procedure. Performing a warm-up sampling during sensory testing has been also found to minimize the first-order carry-over effect [53] and strengthen the reliability of ratings [54,55]. More specifically, the warm-up sample (approximately 45 g), placed in a 118-mL Styrofoam cup (Dart Container Co., Mason, MI, USA) and identified with a three-digit code, was presented at a temperature of approximately 70 °C. Immediately after receiving each sample, participants were asked to rate their willingness to eat the sample and liking of its appearance on 9-point category scales ranging from 1 (“extremely unwilling”/“dislike extremely”) to 9 (“extremely willing”/“like extremely”). Participants were subsequently asked to sniff and rate aroma liking and aroma intensity on two 9-point category scales ranging from 1 (“dislike extremely”/“extremely weak”) to 9 (“like extremely”/“extremely strong”). They were also asked to select all the attribute terms that they considered appropriate for characterizing the aromas of each cooked rice sample using the sensory CATA question ballot. Then, the participants were asked to taste and rate their likings of flavor and mouthfeel on 9-point hedonic scales, and intensities of flavor, sweetness, bitterness, saltiness, and sourness on 9-point category scales ranging from (“extremely weak”) to 9 (“extremely strong”), respectively. The participants were also asked to select all the emotion-related terms that they considered appropriate for describing emotional responses to each cooked rice sample. Finally, they were asked to rate the overall liking and familiarity of each sample on 9-point category scales ranging from 1 (“dislike extremely”/“extremely unfamiliar”) to 9 (“like extremely”/“extremely familiar”).

Following the warm-up sample, five cooked-rice samples, i.e., four test samples and one filler, were presented to each participant in a sequential monadic fashion consistent with the Williams Latin Square design [56]. Participants were asked to evaluate all five samples in the same manner as that for the warm-up sample. A 2-min break was given between sample presentations, and during the break, participants were asked to clean their palate using spring water.

### 2.5. Statistical Analysis

Data were collected using Compusense^®^ five (Release 5.6, Compusense Inc., Guelph, ON, Canada) software and analyzed using SPSS 26.0 for Windows^TM^ (IBM SPSS Inc., Chicago, IL, USA) and XLSTAT software (Addinsoft, Long Island, NY, USA). Data related to the warm-up and filler samples were not used in the statistical analysis.

To determine whether surface colors affected attribute intensity ratings or hedonic ratings of the four cooked-rice samples, a repeated-measures multivariate analysis of variance (RM-MANOVA) was conducted, and univariate repeated-measures analyses of variance (RM-ANOVAs) were conducted if a significant effect was identified [57]. If the sphericity assumption was found to be violated via Mauchly’s sphericity testing, the degrees of freedom were adjusted using a “Greenhouse–Geisser” correction. If a significant effect was found by the RM-ANOVAs, post hoc comparisons between the test samples were performed using Bonferroni *t*-tests. A partial eta-squared (*η*_p_^2^) value was used to measure effect sizes for RM-ANOVA and *η*_p_^2^ values of 0.01, 0.06, and 0.14, respectively, were considered to be small, medium, and large effect sizes [58,59].

A Cochran’s *Q*-test [60] was conducted to determine whether the proportions of selection of individual terms of the sensory or emotion CATA question ballot differed with surface color. If a significant difference among the test samples was found, post hoc multiple pairwise comparisons were conducted using McNemar’s test with Bonferroni alpha adjustment (Bonferroni corrected significance level was 0.0083). To measure the strengths of association between surface colors and specific aroma or emotional attributes selected by participants, Cramér’s *V*-values were used. Cramér’s *V*-values of 0.1, 0.3, and 0.5, respectively, were considered small, medium, and large strengths of association [58,59]. Correspondence analysis was also conducted to visualize associations of surface colors with sensory or emotional attributes of the cooked rice samples [61].

To determine whether associations between surface colors and specific aroma attributes differed as a function of ethnicity-related cultural background (i.e., Caucasian, Asian, or Hispanic), Fisher’s exact tests were performed for each surface color of cooked rice. As a result of the relatively small number (≤2) of participants of other ethnicities, their data were not used in the statistical analysis. Cramér’s *V*-values of 0.1, 0.3, and 0.5, respectively, were considered small, medium, and large strengths of association [58,59]. A statistically significant difference was defined when *p* < 0.05.

## 3. Results

### 3.1. Associations between Surface Colors and Aroma Attributes in Cooked Rice Samples

Table 1 is a contingency table showing the proportions of selection for individual terms listed on the sensory CATA question ballot by participants across the four rice samples. Cochran’s *Q*-test revealed that participants perceived and identified ten aroma attributes of cooked rice samples differently as a function of cooked-rice surface color: cooked rice (*p* = 0.004), ginger (*p* = 0.004), oil (*p* = 0.005), onion (*p* = 0.03), red pepper (*p* < 0.001), saffron (*p* = 0.02), spinach (*p* < 0.001), sweet peas (*p* < 0.001), tomato (*p* < 0.001), and turmeric (*p* < 0.001).

**Figure 2:**
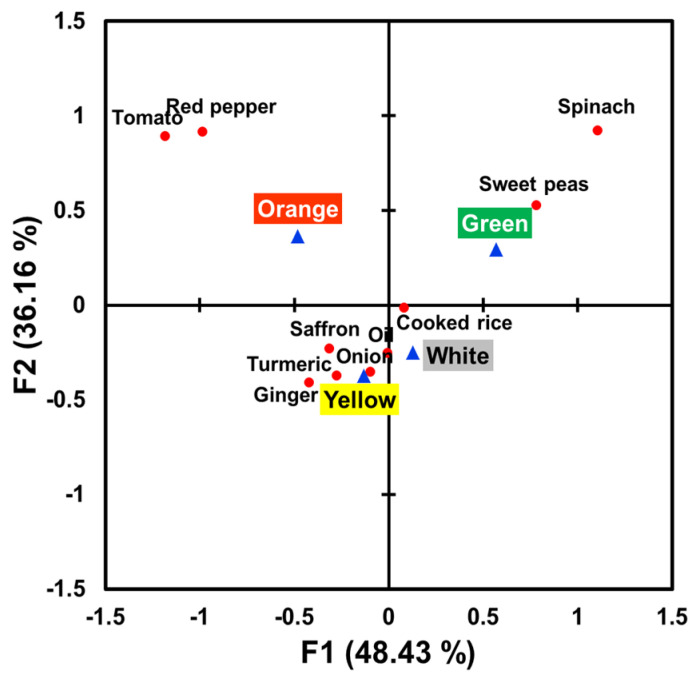
A bi-plot of the correspondence analysis in the associations of color cues (blue triangles) with aroma attributes (red circles) in the cooked rice samples varying in surface colors: white, yellow, orange, and green.

A bi-plot of correspondence analysis (Figure 2), explaining 84.59% of the total variance, visualizes associations between surface colors and aroma attributes. More specifically, while the green color sample was found to be more associated with “sweet peas” and “spinach” aroma attributes, the orange color sample was found to more related to “tomato” and “red pepper” aromas. In addition, while the yellow color sample was found to be more related to “turmeric”, “saffron”, “ginger”, and “oil” aroma attributes, while the white color sample among the four rice samples with different colors was more associated with “cooked rice” aroma.

Multiple pairwise comparison tests using the McNemar (Bonferroni) procedure further clarified the associations between sample colors and aroma attributes. As shown in Table 1, when participants sniffed the green color sample, they perceived a “sweet peas” aroma more frequently than when they sniffed other color samples. Participants also perceived a “spinach” aroma from the green color sample more frequently than from the white or yellow color sample. In addition, when participants sniffed the orange color sample, they reported perceiving “tomato” aroma more frequently than when sniffed other color samples. They also reported perceiving a “red pepper” aroma from the orange color sample more frequently than from the white or green color sample. Moreover, participants perceived a “turmeric” aroma from the yellow color sample more frequently than from the white or green color sample. Although Cochran’s *Q*-test found significant effects of surface color on “ginger”, “oil”, “onion”, and “saffron” aroma attributes, post hoc tests revealed no significant pairwise differences (Bonferroni corrected significance level: 0.0083). Finally, participants perceived a “cooked rice” aroma from the white color sample (i.e., without colorant) more frequently than from the orange color sample.

As shown in Table 2, Fisher’s exact tests revealed significant associations between ethnicity-related cultural background and aroma attributes perceived from each of the cooked rice samples varying in surface color. More specifically, for the white color sample, a proportion of participant selection differed with respect to cloves, oil, or popcorn aroma attributes among the three ethnicity-related cultural backgrounds, i.e., Caucasians, Asians, and Hispanics. For the yellow color sample, the proportion of selection by participants also differed with respect to cilantro, oil, or onion aroma attributes among the three ethnicity-related cultural backgrounds. In addition, with respect to the orange color sample, the proportion of selection differed in oil, onion, or tomato aroma attributes among the three ethnicity-related cultural backgrounds. Overall, participants perceived aroma attributes of ingredients that they would most usually add to their rice meals. For example, Hispanic participants judged that they perceived aroma attributes of other ingredients, i.e., cloves, oil, cilantro, onion, or tomato, which were usually added in their rice meals.

### 3.2. Effect of Surface Colors on Attribute Intensity Ratings of Cooked Rice Samples

**Figure 3:**
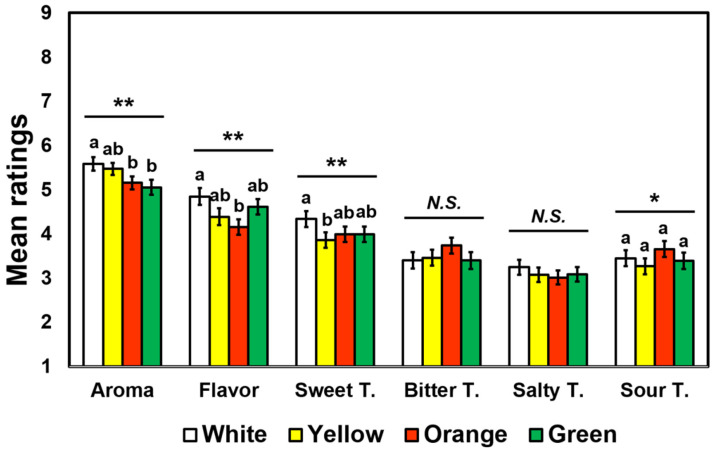
Mean rating comparisons among the four colored cooked-rice samples with respect to intensity ratings of aroma, flavor, sweet taste, bitter taste, salty taste, and sour taste evaluated by 98 participants. Attribute intensities of cooked rice samples were evaluated on 9-point category scales ranging from 1 (extremely weak) to 9 (extremely strong). Error bars represent standard errors of the means. * and ** represent a significant difference at *p* < 0.05 and *p* < 0.01, respectively. Mean ratings with different letters within a category indicate a significant difference determined by post hoc multiple pairwise comparisons Tukey’s Honestly Significant Difference (HSD) tests at *p* < 0.05. *N.S*. represents no significant difference at *p* < 0.05.

Figure 3 shows the mean intensity ratings of aroma, flavor, and four basic taste qualities for the four cooked-rice samples with different surface colors. The RM-MANOVA revealed a significant main effect of surface color on intensity ratings of sensory attributes of cooked rice samples, with a large effect size (*F* = 2.93, *p* < 0.001, Wilks’ lambda = 0.60, *η*_p_^2^ = 0.40). Further univariate RM-ANOVAs revealed significant effects of surface color on intensity ratings of aroma (*F* = 5.03, *p* = 0.002, *η*_p_^2^ = 0.05), flavor (*F* = 5.40, *p* = 0.002, *η*_p_^2^ = 0.05), sweet taste (*F* = 3.87, *p* = 0.009, *η*_p_^2^ = 0.04), and sour taste (*F* = 2.86, *p* = 0.04, *η*_p_^2^ = 0.03); however, post hoc pairwise comparison test revealed no significant difference among the four rice samples with respect to sour taste intensity (*p* > 0.05). More specifically, participants rated the aroma of the white color sample (i.e., without colorant) more intense than that of the orange (*p* = 0.03) or green (*p* = 0.004) color sample. Participants also rated the white color sample more intense with respect to flavor intensity than the orange color one (*p* = 0.002). With respect to sweet taste, participants rated the white color sample more intense than the yellow color sample (*p* = 0.02). However, no significant effects of surface colors were found in the intensity ratings of bitter taste (*p* = 0.08) or salty taste (*p* = 0.19).

### 3.3. Effect of Surface Colors on Hedonic Ratings of Cooked Rice Samples

**Figure 4:**
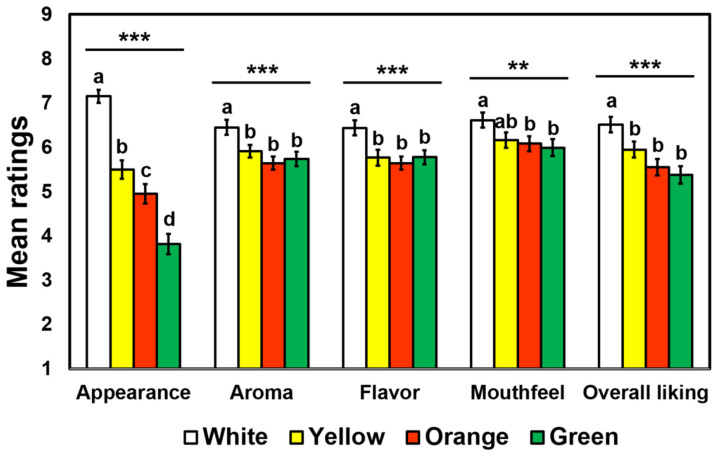
Mean rating comparisons among the four colored cooked-rice samples with respect to hedonic ratings of appearance, aroma, flavor, mouthfeel, and overall liking evaluated by 98 participants. Hedonic impressions of cooked-rice samples were evaluated on 9-point hedonic scales ranging from 1 (dislike extremely) to 9 (like extremely). Error bars represent standard errors of the means. ** and *** represent a significant difference at *p* < 0.01 and *p* < 0.001, respectively. Mean ratings with different letters within a category indicate a significant difference determined by post hoc multiple pairwise comparisons Tukey’s Honestly Significant Difference (HSD) tests at *p* < 0.05.

Figure 4 shows mean hedonic ratings of appearance, aroma, flavor, mouthfeel, and overall impression for the four cooked-rice samples varying in surface color. The RM-MANOVA revealed a significant main effect of surface color on hedonic ratings of cooked rice samples, with a large effect size (*F* = 10.11, *p* < 0.001, Wilks’ lambda = 0.35, *η*_p_^2^ = 0.65). Further univariate RM-ANOVAs revealed significant effects of surface color on hedonic ratings of appearance (*F* = 67.44, *p* < 0.001, *η*_p_^2^ = 0.41), aroma (*F* = 8.57, *p* < 0.001, *η*_p_^2^ = 0.08), flavor (*F* = 6.97, *p* < 0.001, *η*_p_^2^ = 0.07), mouthfeel (*F* = 4.19, *p* = 0.006, *η*_p_^2^ = 0.04), and overall liking (*F* = 11.42, *p* < 0.001, *η*_p_^2^ = 0.11).

Overall, participants gave higher hedonic scores to the white color sample (i.e., without colorant) than other color samples (Figure 4). More specifically, with respect to appearance, participants liked the white color sample the most and the green color sample the least. With respect to mouthfeel, participants liked the white color sample more than either the orange (*p* = 0.04) or the green (*p* = 0.004) color sample.

### 3.4. Associations between Sample Colors and Evoked Emotions toward Cooked Rice Samples

Table 3 is a contingency table showing the proportions of selection by participants across the four rice samples for individual terms listed on the emotion CATA question ballot. Cochran’s *Q*-test revealed that the four cooked rice samples with different colors differed significantly with respect to twelve emotional attributes: “adventure” (*p* = 0.03), “bored” (*p* = 0.02), “glad” (*p* = 0.04), “good” (*p* = 0.001), “mild” (*p* = 0.03), “nostalgic” (*p* = 0.02), “pleased” (*p* < 0.001), “satisfied” (*p* = 0.004), “warm” (*p* = 0.02), “wild” (*p* = 0.003), “friendly” (*p* = 0.01), and “pleasant” (*p* < 0.001).

**Figure 5:**
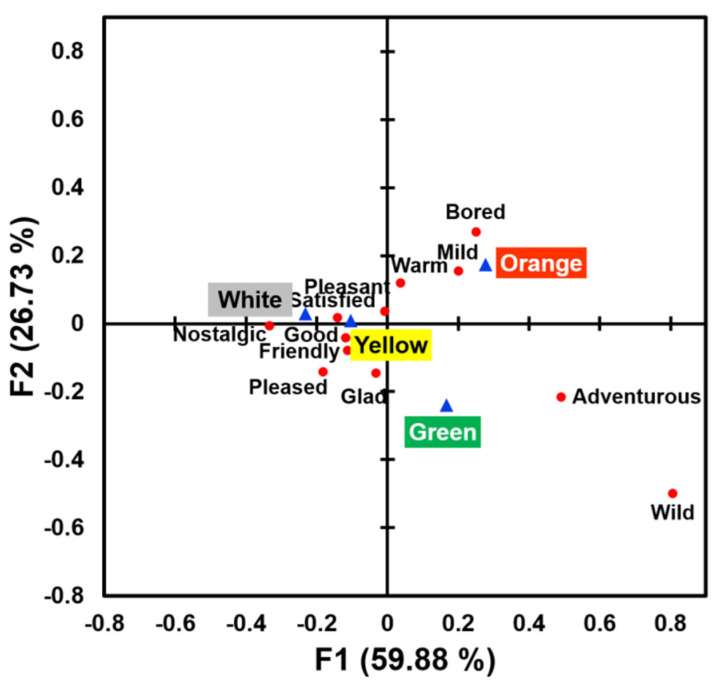
A bi-plot of the correspondence analysis in the associations of color cues (blue triangles) with emotion attributes (red circles) in the cooked rice samples varying in surface colors: white, yellow, orange, and green.

A bi-plot of correspondence analysis (Figure 5), explaining 86.61% of the total variance, reveals associations between surface colors and evoked emotional attributes. More specifically, while the orange color sample was more associated with “bored” and “mild” emotions, the green color sample was more related to “adventurous” and “wild” emotions. In addition, while the yellow color sample was more related to a “friendly” emotion, the white color sample was more associated with “good”, “pleased”, “satisfied”, and “pleasant” emotions.

Multiple pairwise comparison tests using the McNemar (Bonferroni) procedure further revealed how surface colors can be associated with different emotional attributes of cooked rice samples. As shown in Table 3, the “bored” emotion was evoked more frequently in response to the orange color sample than the green color sample, and the “friendly” emotion was more frequently evoked toward the yellow color sample than the orange color sample. While the white color sample more frequently evoked positive emotions such as “good”, “pleased”, “satisfied”, and “pleasant” than the yellow, orange, or green color sample, the green color sample was not exceptional in evoking specific emotional attributes when compared to other color samples.

### 3.5. Effect of Surface Color on Ratings of Willingess to Eat or Familiarity in Cooked Rice Samples

**Figure 6:**
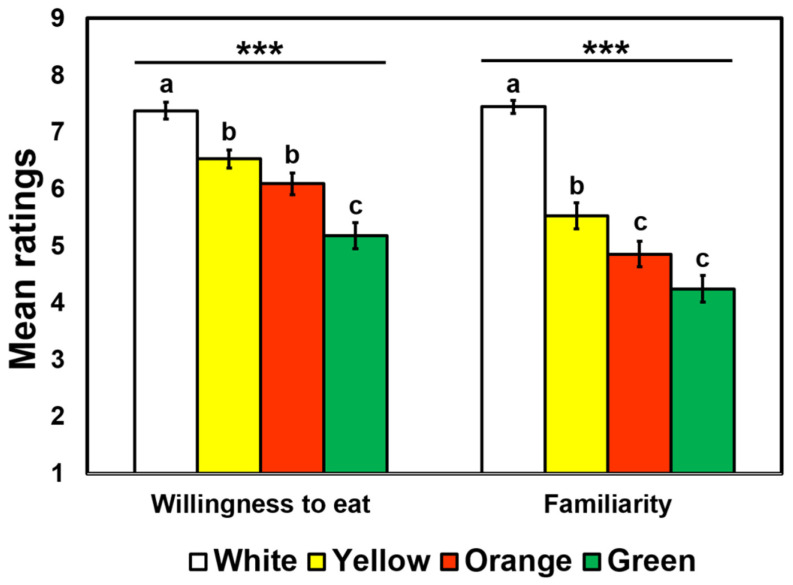
Mean rating comparisons among the four colored cooked-rice samples with respect to willingness to eat and familiarity level evaluated by 98 participants. Willingness to eat and familiarity level of cooked rice samples were evaluated on 9-point category scales ranging from 1 (extremely unwilling/extremely unfamiliar) to 9 (extremely willing/extremely familiar). Error bars represent standard errors of the means. *** represents a significant difference at *p* < 0.001. Mean ratings with different letters within a category indicate a significant difference determined by post hoc multiple pairwise comparisons Tukey’s Honestly Significant Difference (HSD) tests at *p* < 0.05.

Figure 6 shows mean ratings of willingness to eat or familiarity in the four cooked rice samples varying in surface color. A univariate RM-ANOVA found a significant main effect of surface color on ratings of willingness to eat, with a large effect size (*F* = 38.44, *p* < 0.001, *η*_p_^2^ = 0.28). While participants were most willing to eat the white color sample (i.e., without colorant), they were least willing to eat the green color sample. There was also a significant main effect of surface color on ratings of familiarity, with a large effect size (*F* = 76.83, *p* < 0.001, *η*_p_^2^ = 0.44). Similar to the rating trend of willingness to eat, participants rated the white color sample the most familiar, with the orange or green color sample having the least familiarity ratings.

## 4. Discussion

Color cues of food or beverage items provide consumers with anticipatory expectations about what they will experience during their intake. Previous studies have shown that food or beverage items’ color cues can influence consumers’ flavor identification responses (for a review, see [62]). For example, in a study conducted by Zampini et al. [8], eleven female participants were asked to look at each of seven colored drinks (blue, gray, green, red, yellow, orange, and colorless) and then from a list of 22 flavor options identify what flavor they would expect without tasting them (Experiment 1). The results showed that participants expected different flavors corresponding to such color cues. For orange-colored drinks, 91% of participants expected an orange flavor, while for yellow-colored drinks, 89% expected a lemon flavor, and for green-colored drinks, 69% expected a lime flavor. In their subsequent study (Experiment 2) that asked participants to taste and identify the flavors of 28 solution samples varying in flavor (lime, orange, strawberry, or flavorless), color (green, orange, red, or colorless), and amount of food coloring added (standard or double), participants better identified flavors when the solutions were appropriately colored (e.g., orange flavor for orange-colored solutions) than when they were colored inappropriately. Such findings have been also observed in other studies [63,64,65,66]. Building on those previous findings, the results of this study showed that surface colors can lead some people to identify specific aromas related to color cues in the context of cooked rice even though other flavor ingredients had not been added into the cooked rice (Figure 2 and Table 1). For example, participants reported perceiving “tomato” or “red pepper” aroma more frequently from orange-colored cooked rice than from samples of other colors. In other words, participants were likely to match color cues to specific ingredients associated with the colors based on their experiences in everyday life.

The results of this study showed that surface colors can modulate perceived intensities of aroma, flavor, and sweet taste in cooked rice samples (Figure 3). Participants gave higher ratings of attribute intensity (aroma, flavor, and sweet taste) to a white color sample (i.e., without colorant) more than samples of other colors. Lower intensity ratings of attributes were because participants might rate intensities of plausible attributes expected from the color cues. For example, for orange-colored cooked rice, participants were likely to rate intensities of aroma, flavor, and sweet taste for cooked rice that included specific ingredients expected because of color cues (e.g., tomato or red pepper, etc.). In this way, the participants might have given lower ratings because such expected ingredients had not been added to the cooked rice sample. Second, lower intensity ratings of attributes in artificially colored cooked-rice samples are in consistent with previous findings in that participants rated aromas less intense when food samples were less appropriately colored [67] (see also [68]). In other words, because some of the artificially colored cooked-rice samples were inappropriately colored, participants in this study might perceive lower intensities of aromas in those cooked-rice samples. Since there was a strong gap between the expected and actual experiences of artificially colored cooked-rice samples with respect to aroma, flavor, and sweet taste, such suppression (i.e., contrast) effect was observed [69,70]. However, the effect of color cues on intensity ratings of bitterness, saltiness, or sourness in cooked rice samples was not observed (Figure 3), which is not consistent with previous findings related to the association between colors and taste cues [20,21,22,23,24,25,71,72]. The lack of significance of these attributes might be due to their subtle intensities perceived in cooked rice samples [49]. In addition, the discrepancy between the expected and actual perceptions of bitterness, saltiness, or sourness in the colored cooked rice samples might not strong enough to occur such suppression (contrast) effect [69,70]. Previous studies have also found that the effect of color cues on perceived intensities of odors can differ depending on their delivery route. Participants were found to perceive odors more intense when they were smelled by sniffing (i.e., orthonasally) than by tasting (i.e., retronasally) [25,73,74]. In addition, while color cues increased intensity ratings of orthonasal odors, an opposite result was observed when the odors were delivered by tasting (i.e., through retronasal odors) [23]. Previous research has also shown that orthonasal odors of colored solutions were perceived more intense, regardless of whether the colors of the solutions were appropriate or inappropriate, than those of colorless solutions [21,22,23]. In contrast to those findings, our study showed that participants perceived odors as less intense from artificially colored rice samples regardless of the odor’s delivery route (i.e., aroma and flavor), as shown in Figure 3. Thus, further studies should be conducted to elucidate mechanisms underlying the effect of color cues on intensities of aromas or flavors.

Our results disclosed empirical observations that hedonic impressions of cooked rice samples can vary with surface colors. As shown in Figure 4, participants gave higher hedonic ratings to the white color sample than other colored samples, and appearance likings of the green color sample (mean = 3.82) in particular were much lower than those of the white color sample (mean = 7.15). Subsequently, adding colorants decreased hedonic ratings of aroma, flavor, mouthfeel, and overall impression. Such lower likings of yellow, orange, and green color samples with respect to aroma, flavor, and mouthfeel might be associated with disconfirmation between expected and actual perceptions (i.e., contrast effect), resulting in a decrease in hedonic ratings [9,69,70,75,76]. Another plausible explanation for decreased hedonic ratings of the artificially colored cooked rice sample is lower familiarity levels of those samples (Figure 6). Since more than half of participants were Caucasians, even though they were habitual rice consumers, they might be less familiar with such colored cooked rice samples than Hispanic or Asian participants (see Table 2), thereby decreasing hedonic impressions of the colored cooked rice samples. Atypically colored foods were also found to have a negative effect on consumer acceptability [3,19,77].

Surface color cues were found to affect willingness to eat in the context of cooked rice samples. While the white color sample (i.e., without colorant) was most preferred, the green color sample was least preferred in terms of appearance-related willingness to eat. This result was related to familiarity or lack thereof with the colored cooked-rice samples. As shown in Figure 6, the two ratings of “willingness to eat” and “familiarity” showed a similar pattern across the four colored rice samples, and in fact, correlation analysis revealed a significantly positive relationship between the ratings of willingness to eat and familiarity (*r*_392_ = 0.29, *p* < 0.001). Previous studies have also shown that individuals were more likely to eat or purchase items with which they are more familiar [3,36], although that relationship is not always observed [77,78]. In contrast, they were found to be reluctant to try unfamiliar food items (e.g., atypically colored foods) [3,19,77]. This study showed that emotional responses to cooked rice samples can differ with surface color (Figure 5), supporting previous findings associated with variations in emotional responses to color cues of food/beverage items [32,33,34]. While the green color sample was more related to “adventurous” and “wild” emotions, the white color sample was more associated with positive emotions such as “good”, “pleased”, “satisfied”, and “pleasant,” which is in agreement with the respective lower and higher hedonic ratings of the green and white color samples (Figure 4). The orange and yellow color samples were also found to be more associated with “bored” and “friendly” emotions, respectively. This result suggests that product developers, chefs, sensory professionals, and marketers should consider how to coordinate surface colors of meal items in response to emotional states of target consumers. Since food/beverage-evoked emotions have been found to play an important role in consumer acceptability and purchase-related behavior toward food/beverage products [33,34,35,36], it is worth further investigating how to optimize surface color cues for eliciting positive emotional responses to target products [36].

It should be noted that ethnicity-related cultural background influenced associations between color cues and aroma identification. As shown in Table 2, participants reported perceiving aroma attributes of ingredients that they typically add into their rice meals in everyday life. Since many Hispanic people are used to preparing cooked rice meals with oil, cloves, cilantro, onion, or tomato, surface color cues misled them to perceive such ingredient aromas in the cooked rice samples, even the white-colored cooked rice, containing no such additional ingredients. The impact of cultural background on color–flavor association has also been also observed in other studies. In a study by Shankar et al. [9], 20 British and 15 Taiwanese participants reported flavors expected in response to six colored solutions (brown, blue, yellow, orange, green, and red). Significant differences between the two groups in terms of expected flavor were found in brown, blue, yellow, and orange-colored solutions. For example, in response to the brown solution, 70% of British participants expected cola flavor, followed by cherry (15%), and blackcurrant (10%), while 40% of Taiwanese participants expected grape flavor, followed by mulberry (20%) and cranberry (20%). Interestingly, none of British and Taiwanese participants identified “grape” and “cola” flavor, respectively, with the brown solution. Taken together, since cultural background can play an important role in the effect of color cues not only in aroma identification, but also in hedonic impression, willingness to eat (or purchase), and elicited emotions [71,72] (also see [79]), it would be interesting to conduct further studies to assess how the effect of color cues in other rice-consuming countries with different cultural backgrounds, especially countries such as Bangladesh, India, Iran, Spain, etc., where people typically consume colored-rice meals.

The effects of color cues on consumer perception and the liking of food/beverage samples are affected by many factors that have resulted in inconsistent results across previous studies. In addition to the above-mentioned cultural background, gender should be also considered in this regard, because gender effects related to the effects of color cues on perception, liking, and evoked emotions have been exhibited in some studies [27,77,80]. Since age group, education level, and the level of food neophobia have been found to influence consumer responses to food/beverage items with different colors (e.g., typical versus atypical) [77], it would be interesting to determine how such factors can affect consumer perception and behavior toward colored cooked rice samples. The experimental setting (e.g., within-participant design or experimental/task demands) should also be considered a factor related to inconsistency in empirical results related to color–flavor associations [9]. Finally, different dimensions of color cues, other than hues should be considered when drawing general conclusions about the effect of color cues on consumer perception, liking, and evoked emotions, because brightness and saturation of a particular hue can also affect psychological responses to color cues [12,27]. While further studies should be conducted to draw a general mechanism related to the effect of color cues on food perception, the findings of this study provide a better understanding of how to utilize color cues for increasing consumer acceptance and eating-related behavior in the context of cooked rice consumption.

## 5. Conclusions

This study provides empirical evidence that surface color cues affect aroma identification, attribute intensity, and liking, and willingness to eat in the context of cooked rice in which a variety of cooking pattern and dietary habits are extended across cultural backgrounds around the world. Of interest, this study also found that emotional responses to cooked rice can vary with surface color. This study also showed that an ethnicity-based cultural background and familiarity with colored-rice meals can both play a role in modulating the effect of color cues on consumer perception and willingness to eat cooked rice samples that vary in surface colors. Therefore, product developers, sensory professionals, and foodservice professionals should consider cultural background when they design new rice-based meal items.

Further studies, as discussed above, should be conducted to find general mechanisms underlying the effects of color cues on consumer perception, acceptance, evoked emotions, and purchase-related behaviors toward colored cooked rice samples in a wider range of consumer demographic profiles such as ethnicity, cultural background, gender, age group, education level, and food neophobia. While further studies are still needed, the findings of this study can be applied to the design of new rice-based products, regional/cultural background-based product marketing, or sensory nudges designed for modulating not only food perception and acceptance, but also food intake or choice/purchase-related behavior [81,82,83,84].

## Figures and Tables

**Table 1:** A contingency table of the proportions of selection by 98 participants for individual sensory attributes among the four cooked-rice samples with different surface colors.

Attributes	Surface Colors				*p*-Value	Cramér’s *V*-Value ^2^
	White	Yellow	Orange	Green		
Cilantro	0.02 a	0.02 a	0.03 a	0.06 a	0.22	0.09
Cloves	0.03 a	0.01 a	0.03 a	0.02 a	0.72	0.06
Cooked rice	0.95 a ^1^	0.86 ab	0.81 b	0.86 ab	0.004	0.15
Curry	0.01 a	0.08 a	0.05 a	0.04 a	0.08	0.12
Floral	0.13 a	0.08 a	0.09 a	0.15 a	0.15	0.09
Ginger	0.00 a	0.08 a	0.03 a	0.01 a	0.004	0.18
Oil	0.26 a	0.29 a	0.16 a	0.14 a	0.005	0.15
Onion	0.11 a	0.10 a	0.06 a	0.03 a	0.03	0.12
Popcorn	0.21 a	0.19 a	0.15 a	0.13 a	0.17	0.09
Red pepper	0.00 b	0.01 b	0.11 a	0.01 b	<0.001	0.26
Saffron	0.04 a	0.13 a	0.08 a	0.03 a	0.02	0.16
Spinach	0.00 b	0.00 b	0.02 ab	0.12 a	<0.001	0.27
Sweet peas	0.03 b	0.03 b	0.04 b	0.18 a	<0.001	0.25
Tomato	0.00 b	0.02 b	0.19 a	0.00 b	<0.001	0.36
Tumeric	0.02 b	0.19 a	0.07 ab	0.04 b	<0.001	0.25

^1^ Proportions with different letters within a row represent a significant difference at *p* < 0.0083 (Bonferroni corrected significance level). ^2^ Cramér’s *V*-values of 0.1, 0.3, and 0.5, respectively, were considered small, medium, and large strengths of association [58,59].

**Table 2:** A contingency table of the proportions of selection by Caucasian, Asian, and Hispanic participants for individual sensory attributes in the four cooked-rice samples with different surface colors.

Attributes	Ethnicity-Related Cultural Backgrounds			*p*-Value ^2^	Cramér’s *V*-Value ^3^
	Caucasians (*n* = 51)	Asians (*n* = 27)	Hispanics (*n* = 16)		
*White color*					
Cloves	0 (0.0%) ^1^	0 (0.0%)	2 (12.5%)	0.03	0.31
Oil	14 (27.5%)	2 (7.4%)	9 (56.3%)	0.002	0.36
Popcorn	16 (31.4%)	2 (7.4%)	2 (12.5%)	0.03	0.27
*Yellow color*					
Cilantro	0 (0.0%)	0 (0.0%)	2 (12.5%)	0.03	0.31
Oil	14 (27.5%)	4 (14.8%)	9 (56.3%)	0.02	0.30
Onion	4 (7.8%)	1 (3.7%)	5 (31.3%)	0.02	0.31
*Orange color*					
Oil	9 (17.6%)	0 (0.0%)	7 (43.8%)	<0.001	0.38
Onion	3 (5.9%)	0 (0.0%)	3 (18.8%)	0.045	0.25
Tomato	6 (11.8%)	5 (18.5%)	8 (50.0%)	0.005	0.34

^1^ Frequency (% of total Caucasian, Asian, or Hispanic participants); ^2^ *p*-value was obtained by the Fisher’s exact test.; ^3^ Cramér’s *V*-values of 0.1, 0.3, and 0.5, respectively, were considered small, medium, and large strengths of association [58,59].

**Table 3:** A contingency table of the proportions of selection by 98 participants for individual emotional attributes among the four cooked-rice samples with different surface colors.

Attributes	Surface Colors				*p*-Value	Cramér’s *V*-value ^2^
	White	Yellow	Orange	Green		
Active	0.10 a	0.06 a	0.04 a	0.07 a	0.17	0.09
Adventurous	0.04 a	0.07 a	0.11 a	0.13 a	0.03	0.13
Affectionate	0.09 a	0.07 a	0.05 a	0.04 a	0.27	0.08
Aggressive	0.01 a	0.04 a	0.05 a	0.03 a	0.39	0.08
Bored	0.12 ab ^1^	0.13 ab	0.20 a	0.08b	0.02	0.13
Calm	0.30 a	0.24 a	0.26 a	0.21 a	0.46	0.07
Daring	0.01 a	0.03 a	0.03 a	0.07 a	0.08	0.12
Disgusted	0.03 a	0.10 a	0.09 a	0.09 a	0.15	0.11
Eager	0.09 a	0.08 a	0.08 a	0.06 a	0.81	0.04
Energetic	0.12 a	0.09 a	0.10 a	0.13 a	0.68	0.05
Enthusiastic	0.11 a	0.08 a	0.12 a	0.07 a	0.42	0.07
Free	0.09 a	0.07 a	0.09 a	0.06 a	0.69	0.05
Friendly	0.18 ab	0.22 a	0.10b	0.13 ab	0.01	0.13
Glad	0.18 a	0.08 a	0.09 a	0.13 a	0.04	0.12
Good	0.43 a	0.30 ab	0.21b	0.25b	0.002	0.18
Good-natured	0.20 a	0.16 a	0.10 a	0.14 a	0.05	0.10
Guilty	0.00 a	0.04 a	0.02 a	0.01 a	0.14	0.11
Happy	0.20 a	0.18 a	0.17 a	0.18 a	0.93	0.03
Interested	0.22 a	0.28 a	0.27 a	0.29 a	0.66	0.05
Joyful	0.16 a	0.11 a	0.09 a	0.09 a	0.18	0.09
Loving	0.13 a	0.07 a	0.06 a	0.07 a	0.15	0.10
Merry	0.08 a	0.05 a	0.06 a	0.04 a	0.46	0.06
Mild	0.24 a	0.16 a	0.29 a	0.16 a	0.03	0.13
Nostalgic	0.14 a	0.09 a	0.04 a	0.05 a	0.02	0.15
Peaceful	0.22 a	0.17 a	0.17 a	0.16 a	0.50	0.06
Pleasant	0.38 a	0.27 ab	0.19b	0.18b	<0.001	0.18
Pleased	0.33 a	0.18 ab	0.11b	0.19 ab	<0.001	0.19
Polite	0.10 a	0.09 a	0.07 a	0.12 a	0.51	0.06
Quiet	0.12 a	0.16 a	0.17 a	0.10 a	0.26	0.08
Satisfied	0.38 a	0.20b	0.26 ab	0.21b	0.004	0.16
Secure	0.11 a	0.07 a	0.05 a	0.07 a	0.24	0.08
Steady	0.06 a	0.11 a	0.14 a	0.09 a	0.16	0.10
Tame	0.00 a	0.03 a	0.05 a	0.01 a	0.07	0.13
Tender	0.08 a	0.09 a	0.06 a	0.07 a	0.78	0.04
Understanding	0.13 a	0.12 a	0.07 a	0.13 a	0.28	0.08
Warm	0.31 a	0.19 a	0.26 a	0.16 a	0.02	0.13
Whole	0.11 a	0.09 a	0.05 a	0.04 a	0.08	0.11
Wild	0.00 a	0.02 a	0.05 a	0.08 a	0.003	0.16
Worried	0.01 a	0.05 a	0.07 a	0.06 a	0.15	0.11

^1^ Proportions with different letters within a row represent a significant difference at *p* < 0.0083 (Bonferroni corrected significance level). ^2^ Cramér’s *V*-values of 0.1, 0.3, and 0.5, respectively, were considered small, medium, and large strengths of association [58,59].
